# Situational and Victim Correlates of Increased Case Fatality Rates in Los Angeles Shootings, 2005–2021

**DOI:** 10.1007/s11524-024-00845-z

**Published:** 2024-03-28

**Authors:** P. Jeffrey Brantingham, Miguel Quintana-Navarrete, Clarissa Iliff, Craig D. Uchida, George E. Tita

**Affiliations:** 1 Department of Anthropology, University of California, Los Angeles, 341 Haines Hall, Los Angeles, CA 90095 USA; 2 Department of Criminology, Law & Society, 2309 Social Ecology II, Irvine, CA 92697 USA; 3 Justice & Security Strategies, Inc., PO Box 6188, Silver Spring, MD 20916 USA

**Keywords:** Gun violence, Firearm deaths, Crime data, Demography, United States

## Abstract

The gun assault case fatality rate measures the fraction of shooting victims who die from their wounds. Considerable debate has surrounded whether gun assault case fatality rates have changed over time and what factors may be involved. We use crime event data from Los Angeles to examine the victim and situational correlates of gun assault case fatality rates over time. We estimated log binomial regression models for the probability of death in each year from 2005 to 2021, conditioned on situational and victim characteristics of the crime. Case fatality rates increased by around 1.3% per year between 2005 and 2021 from around 15.9 to 19.7%. Baseline case fatality rates differed systematically by most situational and victim but followed similar temporal trends. Only victim age significantly covaried with the temporal trend in case fatality rates. An individual shot in Los Angeles in 2021 was 23.7% more likely to die than the equivalent victim in 2005. The steady increase in case fatality rates suggests that there were around 394 excess fatalities over what would have occurred if case fatality rates remained at the 2005 level. Increases in the average age of victims over time may contribute to the general temporal trend. We hypothesize that older victims are more likely to be shot indoors where lethal close-range wounds are more likely.

## Introduction

Many American cities experienced a worrying spike in homicides in 2020 and 2021 [1, 2]. The headlines have usually been accompanied by a caveat that homicide numbers remain well below the extremes of the early 1990s [3]. Indeed, based on the FBI’s Expanded Homicide Data [4], the 17,815 homicides recorded nationally in 2020 was 44% *above* the low point of 12,312 homicides in 2014 but also 23% *below* the 23,225 homicides recorded in 1993. However, homicides should not be viewed in isolation, but as one extreme outcome of violence. Thus, in this paper, we take a more expansive approach to the study of homicides by examining the gun assault case fatality rate—or the fraction of shooting victims who die from their wounds—and its relationship to broader patterns and processes in violent crime victimization.

A few studies have adopted this broader perspective using data sourced from either health care or crime event settings. Brantingham et al. [5], for example, used emergency room and hospital admissions data to show that the number of victims of gun assaults declined in California between 2005 and 2019 alongside homicides but the case fatality rate increased steadily over this 15-year period. The mechanisms driving increased case fatality rates were not addressed, however. Jena et al. [6] used data from the CDC to compare non-fatal and fatal gun assaults nationally, finding that the former increased between 2002 and 2011 while the latter remained relatively stable (see also [7]). Case fatality rates thus declined over this period. Cook et al. [8] in response to the findings of Jena et al. used data from the National Electronic Injury Surveillance System (NEISS) and CDC to reexamine case fatality rates for gun assaults for the period 2003–2012. Their concern was to correct for the effects of hospital substitutions over time in the dataset and a decline in the number of events listed as “unknown circumstance.” Both processes may introduce a downward bias in estimates of case fatality rates. Making reasonable assumptions, Cook et al. [8] concluded that there was no change in gun assault case fatality rates over this 10-year period.

Kaufman et al. [9] took a different approach and compared case fatality rates recorded in both trauma center and police data for Philadelphia, PA, from 2005 to 2014. Both data sets painted a picture of declining numbers of gun assaults over time. However, the two data sets were inconsistent in what they showed. Trauma center data suggested substantial declines in case fatality rates, while the police data showed no significant change over time. The results aligned well with the conclusions of Cook et al. [8]. The authors explained the discrepancy by pointing out that trauma center data tend to undercount gun assaults and overestimate mortality. Police data, by contrast, include many gun assaults where individuals are wounded, but victims are treated either at the scene or in non-trauma medical facilities. Police data also may be more likely to include homicides where the individual died at the scene and therefore never appeared in trauma center records. Kaufman et al. [9] suggested that police records “are likely to give a more accurate view of firearm injury epidemiology and changes over time, although without the clinical depth of [trauma center data].” This perspective echoed Cook [10] who regarded police records as an appropriate source to estimate the “true death rate from gunshot wounds.”

Focusing not on trends but on potential causes, Cook et al. [11] used emergency department admission data to examine the effect of age on case fatality rates among youth. Using multilevel mixed-effects regression, they found that the probability of dying was highest among the youngest victims (< 5 years old) and that the probability declines sharply with age (up to a maximum of 19 years old). Shifting the focus to race/ethnicity, Cook, Osler et al. [12] found that Black victims of gun assaults were admitted to hospitals at higher rates than any other racial-ethnic group, but also that the post-admission fatality rates for Blacks did not differ significantly from non-Hispanic Whites. Braga and Cook [13] turned their attention to the nature of weaponry. They presented a cross-sectional study of the effect of gun caliber on the probability of death for criminal assault victims. Using case files from the Boston Police Department for 2010–2014, they found that the caliber of the gun did not have an effect on the number of wounds but did impact the probability of victim death. Medium caliber weapons increased the odds of dying by a factor of 2.25 and large caliber weapons by a factor of 4.54 relative to small caliber weapons.

The takeaway from these studies is that any general temporal trend in case fatality rates over the past two decades is not clear, nor is there a clear understanding of the mechanisms at play in driving change (or the lack thereof) in case fatality rates over time. The present study uses crime event data to document changes in the gun assault case fatality rates in the City of Los Angeles for the 17-year period from 2005 to 2021. Though specific to a particular region, the data provide a long-term perspective on the changing nature of interpersonal gun violence. Crime event data is also collected with investigatory goals in mind, which translates into a suite of unique covariates, not typically present in hospital data, that may help clarify the roles that situational conditions and victim characteristics play in making gun violence such a challenging public health problem [9, 13, 14].

## Methods

We examined all fatal and non-fatal gun assaults known to the Los Angeles Police Department (LAPD) for the period from 2005 to 2021. The data from 2010 to 2021 are publicly available (see https://data.lacity.org). The data covering the five years from 2005 to 2009 were provided by the LAPD. We used a combination of “modus operandi” (MO), crime type (CT), and premise type (PT) codes to partition and label the data. Each event is associated with one or more MO codes. Thus, from the larger dataset, we extracted all crimes where the victim was physically shot (MO 0430). From this subset of the data, we then extracted all aggravated assaults (CT 230), robberies (CT 210), and attempted robberies (CT 220), which together represent non-fatal gun assaults (coded as 0). We also extracted homicides (CT 110), which represent fatal gun assaults (coded as 1). MO codes were used to further identify gang-related (MO 0906) [15] and drive-by shootings (MO 0309). The LAPD manual (§269.10) states that “Any crime may constitute a gang-related crime when the suspect or victim is an active or affiliate gang member, or when circumstances indicate that the crime is consistent with gang activity” [16]. PT codes were used to identify events that occurred outdoor (PT 100–199) compared with indoor contexts (all other PT codes). The data included the race-ethnicity, gender, and age of each victim as well as temporal and geographic information.

We used a log binomial regression [17] to estimate the probability $\widehat{p}$ that a shooting produced a homicide. This measure is equivalent to the case fatality rate for the population of crimes. We proceed incrementally by first estimating a baseline model focused only on the temporal trend and then gradually adding covariates to provide insights into the potential roles of different situational and victim characteristics. We present our results as risk ratios that can be interpreted as the percent change in case fatality rate per unit increase in a covariate. All analyses were performed in Stata 17.0.

## Results

**Fig. 1:**
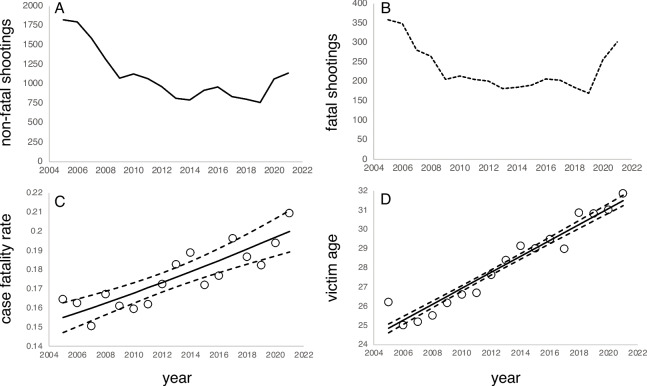
**A** Number of non-fatal shootings (assaults and robberies) in Los Angeles 2005–2021. **B** Number of fatal shootings (homicides). **C** Observed and predicted CFR (Model 3). **D** Observed and predicted victim age for gun assaults over time

Over the 17-year period from 2005 to 2021, there were 18,815 non-lethal gun assaults (assaults and robberies) with a victim shot and 3963 lethal gun assaults (homicides) in the city of Los Angeles (Table 1). The numbers of both event types declined steadily through 2014, experienced a slight increase in 2015–2016, and reached a minimum for the entire 17-year period in 2019 (Fig. 1 A and B). In 2020 and 2021, both non-lethal and lethal gun assaults showed sharp increases. Latino victims outnumbered victims in other ethno-racial groups, representing 50.6% of all non-fatal shootings and 51.6% of all gun homicides over the period. Black victims represented 43.4% of all non-lethal gun assaults and 40.6% of all lethal gun assaults. White and Asian victims, respectively, comprised 2.9% and 0.4% of non-lethal and 4.5% and 0.8% of lethal gun assaults. The proportion of Latino victims of fatal and non-fatal gun assaults combined was similar to their share of the population (48.1%). The proportion of Black victims exceeded their share of the population (8.78%) by a factor of 5.8. The proportions of White and Asian victims fell below their share of the population (28.5% and 11.8%) by factors of 9.1 and 22.4, respectively. Female victims represented 11.0% of the non-fatal gun assaults and 9.6% of the fatal gun assaults. Male, victims made up 88.6% and 90.3% of non-fatal and fatal gun assaults, respectively.

**Table 1:** Counts of fatal and non-fatal gun assaults by victim and situational characteristics and year in Los Angeles, CA 2005–2021

	Non-fatal gun assault (*N*) †	Fatal gun assault (*N*) ‡	Total (*N*)	CFR
White	538	177	715	0.24
Black	8190	1609	9799	0.16
Latino	9548	2044	11,592	0.17
Asian	79	33	112	0.29
Other	496	100	596	0.16
Female	2082	380	2462	0.15
Male	16,699	3580	20,279	0.17
Other/unknown	70	3	73	0.04
Non-gang	6032	1129	7161	0.15
Gang-related	12,819	2834	15,653	0.18
Non-driveby	15,884	3607	19,491	0.19
Driveby	2967	356	3323	0.13
Indoor	3501	921	4422	0.2
Outdoor	15,350	3042	18,392	0.16
2005	1821	359	2180	0.16
2006	1798	349	2147	0.16
2007	1584	281	1865	0.15
2008	1320	265	1585	0.16
2009	1072	206	1278	0.16
2010	1127	214	1341	0.15
2011	1066	206	1272	0.16
2012	965	201	1166	0.17
2013	814	182	996	0.18
2014	794	185	979	0.18
2015	919	191	1110	0.17
2016	963	207	1170	0.17
2017	835	204	1039	0.19
2018	806	185	991	0.18
2019	763	170	933	0.18
2020	1064	256	1320	0.19
2021	1140	302	1442	0.21
Total	18,851	3963	22,814	0.17

^†^ includes only robberies and aggravated assaults where a victim is non-fatally shot; ‡ incudes only robberies and aggravated assaults where the victim is fatally shot

Moving to the attributes of the events, gang-related crimes accounted for 68.0% (*n* = 12,577) of non-lethal and 71.5% of lethal gun assaults (Table 1). Most shootings were *not* drive-bys; the latter accounted for only 15.7% (*n* = 2967) of non-lethal and 9.0% (*n* = 356) of lethal gun assaults. The majority of shootings occurred in outdoor settings, accounting for 81.4% (*n* = 15,350) of non-lethal and 76.8% (*n* = 3,042) of lethal gun assaults.

### Case Fatality Rates Over Time

Case fatality rates increased between 2005 and 2021 (Fig. 1C). This trend is captured across all regression models (Table 2). Model 1, which includes only time as a predictor, indicates that the estimated case fatality rate increased by around 1.6% per year from 15.5% in 2005 to 20.0% in 2021.

**Table 2:** Estimated effects of situational conditions on CFR

Variable	Model 1		Model 2		Model 3		Model 4	
	RR (SE)	*Z*	RR (SE)	*Z*	RR (SE)	*Z*	RR (SE)	*Z*
Year	1.016 (0.003)	5.72***	1.014 (0.003)	5.0***	1.015 (0.003)	5.11***	1.006 (0.003)	2.14*
Gang-related			1.25 (0.042)	6.84***	1.278 (0.043)	7.3***	1.397 (0.048)	9.67***
Drive-by			0.59 (0.031)	− 9.98***	0.596 (0.032)	− 9.72***	0.6 (0.032)	− 9.52***
Outdoor			0.82 (0.028)	− 5.81***	0.821 (0.029)	− 5.66***	0.85 (0.03)	− 4.57***
Race-ethnicity†								
Black					0.659 (0.046)	− 5.99***	0.703 (0.051)	− 4.9***
Latino					0.702 (0.048)	− 5.13***	0.788 (0.056)	− 3.34***
Asian					1.143 (0.182)	0.84	1.14 (0.188)	0.8
Male‡					1.137 (0.057)	2.56**	1.171 (0.061)	3.01**
Victim age§								
15–19							1.632 (0.274)	2.92**
20–24							1.861 (0.311)	3.72***
25–29							2.238 (0.375)	4.81***
30–34							2.337 (0.395)	5.02***
35–39							2.577 (0.44)	5.55***
40–44							2.881 (0.496)	6.15***
45–49							2.811 (0.495)	5.87***
50–54							2.541 (0.465)	5.1***
55–59							3.108 (0.592)	5.95***
60–64							2.787 (0.604)	4.73***

^†^, white is held out the reference category, “other race-ethnicity” excluded; ‡, female is held out as the reference category; §, 10–14 age group held out as reference category; *p < 0.05, **p < 0.01, ***p < 0.001

### Event Characteristics

Model 2 adds situational variables to the baseline model. Model 2 shows that gang-related gun violence was significantly more deadly than non-gang gun violence, while drive-by shootings and gun assaults occurring outdoors were both significantly less deadly (Table 2). LAPD identifies a crime as “gang-related” when the suspect or victim is an active or affiliate gang member, or when circumstances indicate that the crime is consistent with gang activity [15, 16]. An average gang-related gun assault was 25% *more likely* to result in death than an average non-gang gun assault. By contrast, a victim shot in a drive-by shooting was 41% *less likely* to die than in other shootings. Controlling for all other factors, outdoor gun assaults were 18% *less likely* to result in death.

### Victim Characteristics

Model 3 adds race-ethnicity and sex as covariates. The temporal trend in case fatality rates and the impact of situational characteristics remain qualitatively similar after controlling for the race-ethnicity and sex of victims (Table 2). Case fatality rates were higher for Asian (not significant) and White victims compared to Black and Latino victims. Specifically, the risk of dying from a gunshot was 34.1% *lower* for Black victims and 29.8% *lower* for Latino victims compared with White victims, the reference group. Case fatality rates were 13.7% higher for male compared with female victims.

The higher case fatality rates for Asian and White victims compared with Black and Latino victims in part reflect situational differences in gun assaults associated with these groups. Shootings involving Asian and White victims were determined to be gang-related events in 41.1% (*n* = 46) and 35.5% (*n* = 254) of cases, respectively, compared with 70.1% (*n* = 6930) and 70.4% (*n* = 8158) for Black and Latino victims, respectively. Asian and White victims were involved in drive-by shootings 0.9% (*n* = 42) and 5.9% (*n* = 42) of the time, respectively, while Black and Latino victims were involved in drive-by shootings 16.7% (*n* = 1635) and 13.8% (*n* = 1600) of the time, respectively. Asian and White victims were shot outdoors 61.7% (*n* = 69) and 65.5% (*n* = 468) of the time, respectively, compared with 80.6% (*n* = 7894) and 82.3% (*n* = 9542) for Black and Latino victims, respectively. The prevalence of non-gang and non-drive-by shootings combined with indoor settings is associated with greater lethality for Asian and White victims. Conversely, the prevalence of gang-related and drive-by shootings combined with outdoor settings translates into lower lethality for Black and Latino victims.

Model 4 adds victim age (Table 2). The analyses were restricted to victims between the ages of 10 and 65, representing 97.5% (*n* = 22,238) of all lethal and non-lethal gun assaults between 2005 and 2021. The models failed to converge when including victims of all ages due to the sparsity of observations for very young and very old ages. Case fatality rates increased linearly for victims aged 10 to 45, with a slower increase for older victims. Victims between the ages of 15 and 19 were 63% more likely to die from a gunshot than victims aged 10–14, the reference group. Victims aged 40–45 were 188% more likely to die than victims aged 10–14. Victims aged 60–64 were 179% more likely to die compared to the reference group.

**Fig. 2:**
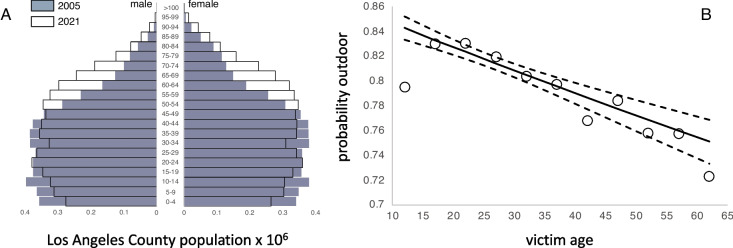
**A** Estimated population proportion in 5-year age groups in 2005 and 2021 for Los Angeles County. Data from the California Department of Finance [19]. **B** Probability that a shooting (non-fatal and fatal combined) occurs outdoors as a function of the victim age

Importantly, the temporal trend in case fatality rates was reduced to just 0.6% per year when victim age was included in the model (Table 2). The offset reflects an underlying increase in the average age of gun assault victims between 2005 and 2021 (Fig. 1D). In 2005, the expected age of a gun assault victim was 24.9 years old, for victims aged 10–65. By 2021, the expected age had shifted to 31.5 years old. The trend is consistent with a recent analysis of arrest data demonstrating a precipitous drop in youth arrests for violent crime [18]. It also tracks an increase in the mean age of the population in Los Angeles County overall (Fig. 2A), which jumped from 33.8 years in 2005 to 39.2 years in 2021 [19]. The mean age for Black and Latino subpopulations in Los Angeles increased from 34.6 to 40.3 years and 27.8 to 35.0 years, respectively. The results suggest that the character of gun violence victimization in Los Angeles over this period was influenced by a demographic shift in the pool of potential victims.

## Discussion

Gun assault case fatality rates in Los Angeles increased significantly between 2005 and 2021. Using Model 3, which excludes victim age, every 100 victims shot in 2005 produced around 16 homicides. By 2021, every 100 victims shot produced around 20 homicides. Despite the decline in overall numbers of shootings, the increase in case fatality rates produced a substantial number of additional homicides. For example, if the case fatality rate estimated by Model 3 for 2005 ($\widehat{p}=0.157$) had held over the entire 17-year period, there would have been around 394 fewer homicides than actually occurred (Table 1). The estimated excess number of fatal gun assaults is more than the yearly average number of gun homicides over the period ($\overline{n }=233.1)$. The general temporal trend corroborates the pattern observed for California as a whole using hospital data [5].

There were also significant differences in case fatality rates by key situational and victim characteristics. Case fatality rates were higher for gang-related crimes, but lower for drive-by shootings and those that occurred outdoors. Case fatality rates were higher for Asian and White victims of gun assaults, compared with Black and Latino victims, and higher for males compared with females. The temporal trend seen in Model 3 is associated with a parallel increase in average victim age, which carries through to the estimated case fatality rates seen in Model 4. There are several reasons why a shift to older victims might lead to higher case fatality rates. First, the number and severity of comorbidities generally increase with age [20], which may mean that older individuals are more likely to die from a gunshot wound, all else being equal. Second, prior studies suggest that higher caliber guns result in a higher probability of dying if shot [13]. If victim age is somehow associated with the caliber of gun used, then the shift towards older victims may indirectly cause a shift in the case fatality rates through gun caliber. Finally, we observe that victim age is negatively associated with the probability that a shooting occurs outdoors (Fig. 2B). In other words, older individuals more often find themselves the victims of shootings indoors compared with outdoors perhaps because a greater share of interactions with close family or friends occur indoors. We conjecture that these indoor shootings may be more lethal because the tighter spatial quarters produce closer contact with a greater chance of head or torso injury [21, 22]. Unfortunately, we are unable to evaluate any of these hypotheses with the present data.

This study is limited in several respects. We are only able to examine events known to the police. Reporting rates for lethal and non-lethal shootings with a victim hit are typically much higher (90%) than for most other crime types (≤ 50%) [23]. Underreporting is likely to influence non-fatal gun assaults more than gun homicides, which could artificially inflate case fatality rates. However, crime underreporting rates appear to be stable over time [24] suggesting that a long-term shift in crime reporting does not explain the temporal trend.

In conclusion, both non-fatal and fatal shootings have fallen in number over the past 17 years in Los Angeles. However, the decline has been steeper for non-fatal shootings resulting in a steady increase in case fatality rates. California, like much of the United States, is projected to get much older in the coming decades [25, 26]. To the extent that victim age is a reliable predictor of the probability of dying when shot, the present study suggests that gun violence case fatality rates may continue to climb along with the aging population. While there appears to be limited policy room to steer demography [27], policies that target the convergence of age and the situational factors of gun assaults may be worth pursuing. For example, hot spot policing [28], focused deterrence [29], civilian violence interruption programs [30], or the greening of public spaces [31] might make use of age information to better target interventions towards older subpopulations. Efforts to reduce certain high-risk behaviors such as unsafe gun storage in the home could target older individuals, with the expectation that fatal gun assaults might be disproportionately affected [32].

## Data Availability

Los Angeles crime data is publicly available for all years beginning with 2010 (see https://data.lacity.org). The publicly available data are sufficient to reproduce qualitatively similar results. Data from 2015–2009 may be requested by contacting the Los Angeles Police Department.
